# Non-coding RNAs in skin cancers:Biological roles and molecular mechanisms

**DOI:** 10.3389/fphar.2022.934396

**Published:** 2022-08-10

**Authors:** Fei Liu, Shenglong Li

**Affiliations:** Department of Bone and Soft Tissue Tumor Surgery, Cancer Hospital of China Medical University, Liaoning Cancer Hospital & Institute, Shenyang, China

**Keywords:** skin cancers, non-coding RNA, malignant progression, molecular biology, metastasis

## Abstract

Cutaneous malignancies, including basal cell carcinoma, cutaneous squamous cell carcinoma, and cutaneous melanoma, are common human tumors. The incidence of cutaneous malignancies is increasing worldwide, and the leading cause of death is malignant invasion and metastasis. The molecular biology of oncogenes has drawn researchers’ attention because of the potential for targeted therapies. Noncoding RNAs, including microRNAs, long noncoding RNAs, and circular RNAs, have been studied extensively in recent years. This review summarizes the aspects of noncoding RNAs related to the metastasis mechanism of skin malignancies. Continuous research may facilitate the identification of new therapeutic targets and help elucidate the mechanism of tumor metastasis, thus providing new opportunities to improve the survival rate of patients with skin malignancies.

## 1 Introduction

**FIGURE 1:**
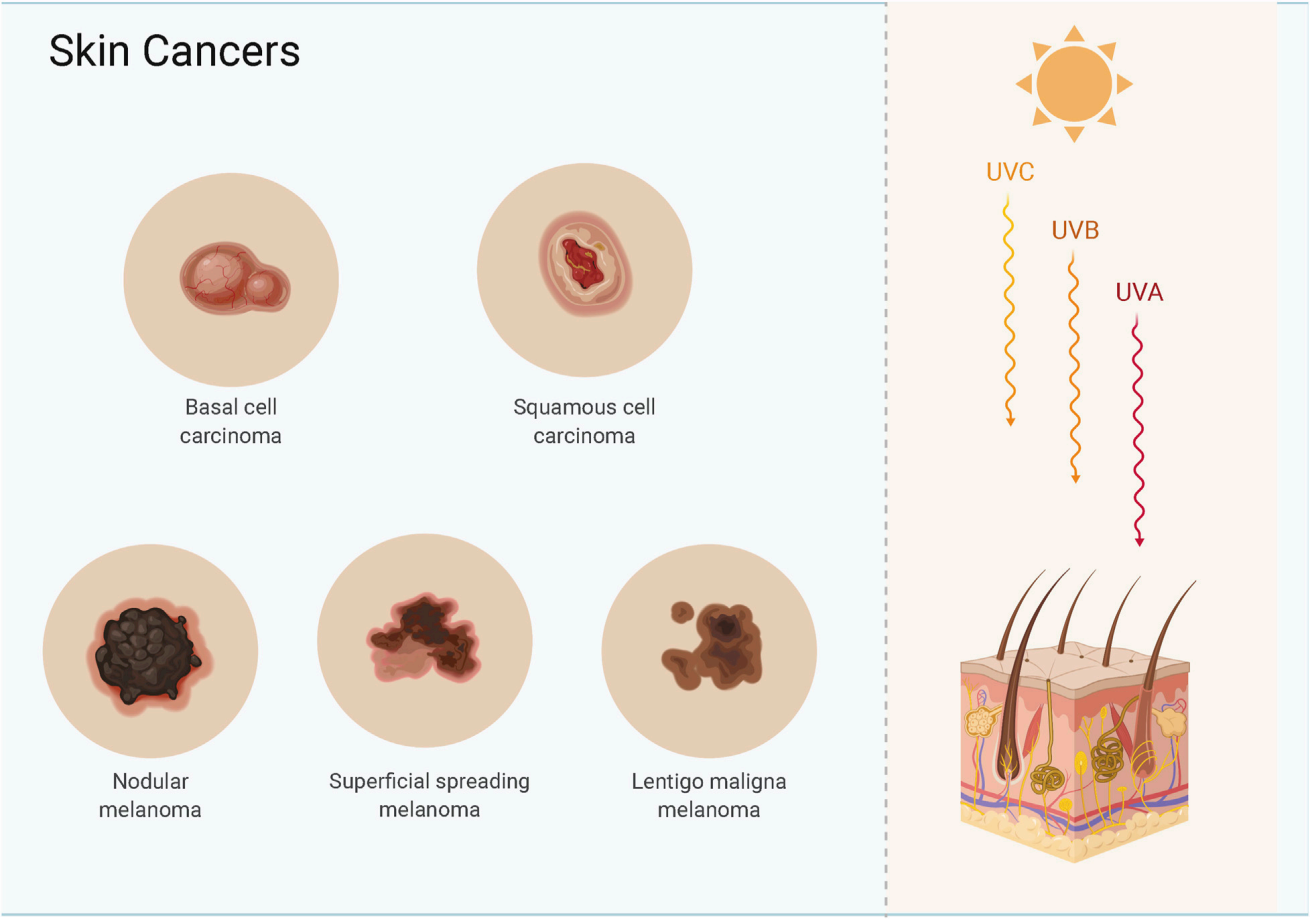
Skin cancers include basal cell carcinoma, cutaneous squamous cell carcinoma and melanoma.

Skin cancer is a common malignancy, and the most common types are basal cell carcinoma (BCC), cutaneous squamous cell carcinoma (CSCC), and cutaneous malignant melanoma (CMM) (Sato et al., 2021) (Figure 1). BCC and CSCC are more prevalent than melanoma, and most patients receive prompt treatment leading to a better long-term prognosis (Bednarski et al., 2021; Reddy et al., 2021). BCC and CSCC originate from epidermal keratin-forming cells and have a lower mortality than melanoma; the lesion is confined to the site of origin, and the treatment is thus straightforward (Cives et al., 2020; Krasowska et al., 2021). CMM is a difficult-to-treat metastatic malignancy that originates from epidermal melanocytes and is associated with a high mortality (Fujimura and Aiba 2020). When detected early, melanoma is treatable by surgical excision; however, rapid invasion and metastasis are the main reasons for the lower survival time after advanced treatment (Allegra et al., 2020). Genetic changes can induce the transformation of normal cells into cancer cells, and cancer cells can become malignant after cell division**.**

Noncoding RNAs (ncRNAs) include microRNAs (miRNAs), long noncoding RNAs (lncRNAs), and circular RNAs (circRNAs) (He et al., 2021; Mercer et al., 2022). NcRNAs perform their respective biological functions at the RNA level (Xiaoqing Li et al., 2021). NcRNAs bind to various molecular targets to initiate specific cellular responses and regulate gene expression, intracellular signaling, epigenetic modifications, and other functions. The ncRNA is vital in the occurrence and development of tumors including skin cancer (Ding et al., 2021; Winkle et al., 2021). Accumulating evidence highlights the importance of ncRNAs in skin cancer. For example, circRNA_0016418 cross-talks with miR-625 and suppresses the progression of human skin melanoma (Zou et al., 2019). LncRNA MEG3 acts as a sponge for miR-21 and promotes melanoma growth and metastasis (Wu et al., 2020). The function of ncRNAs in cutaneous cancers was described previously (Zhou et al., 2022). Here, we provide an update of recent findings on the role of ncRNAs in skin cancer.

## 2 Noncoding RNAs in cancer

### 2.1 MiRNAs

**FIGURE 2:**
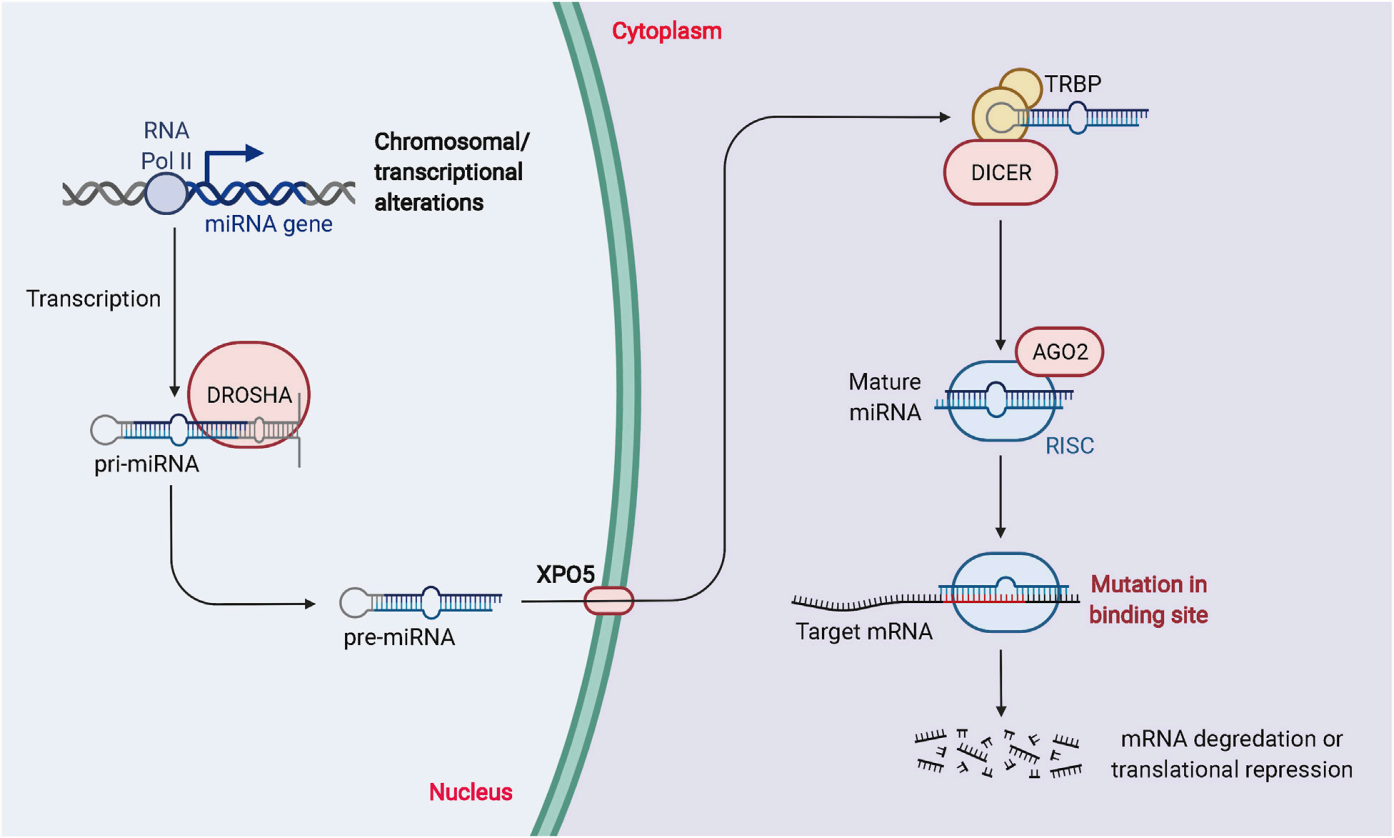
The biogenesis and biological function of miRNAs. RNA Pol II or III can regulate the transcription of pri-miRNAs, and DROSHA and DGCR8 can process the nuclear processing of pri-miRNAs into pre-miRNAs. Exportin 5 is involved in the processing of pre-miRNAs for nuclear export. Dicer and TRBP modulation regulate the cytoplasmic processing of pre-miRNAs into mature miRNA duplexes. miRNA duplexes include passenger strands and mature miRNAs. The passenger strand is degraded, and the mature miRNA strand is integrated into RISC to mediate translational repression or mRNA degradation depending on the degree of complementarity to the mRNA target.

MiRNAs are essential ncRNAs that regulate protein biosynthesis by modulating transcription (Kakumani 2022; Singh et al., 2022). The synthesis of miRNAs is catalyzed by RNA polymerase II, which produces primary miRNAs that enter the nucleus. Drosha, a family of ribonucleases, further catalyzes the production of precursor miRNAs with a hairpin structure (Asakiya et al., 2022; Quirico et al., 2022). This double-stranded product consists of a mature miRNA guide strand and a miRNA guest strand. Both miRNAs can be loaded into the RNA-induced silencing complex to degrade or inhibit translation of mRNA, thereby affecting protein expression (Shunhao Zhang et al., 2021; Das et al., 2022; Diener et al., 2022). A single miRNA can target hundreds of mRNAs, affecting the expression and interactions of many genes and participating in the regulation of numerous physiological and pathological processes. The biogenesis and biological function of miRNAs is described in Figure 2.

### 2.2 LncRNAs

**FIGURE 3:**
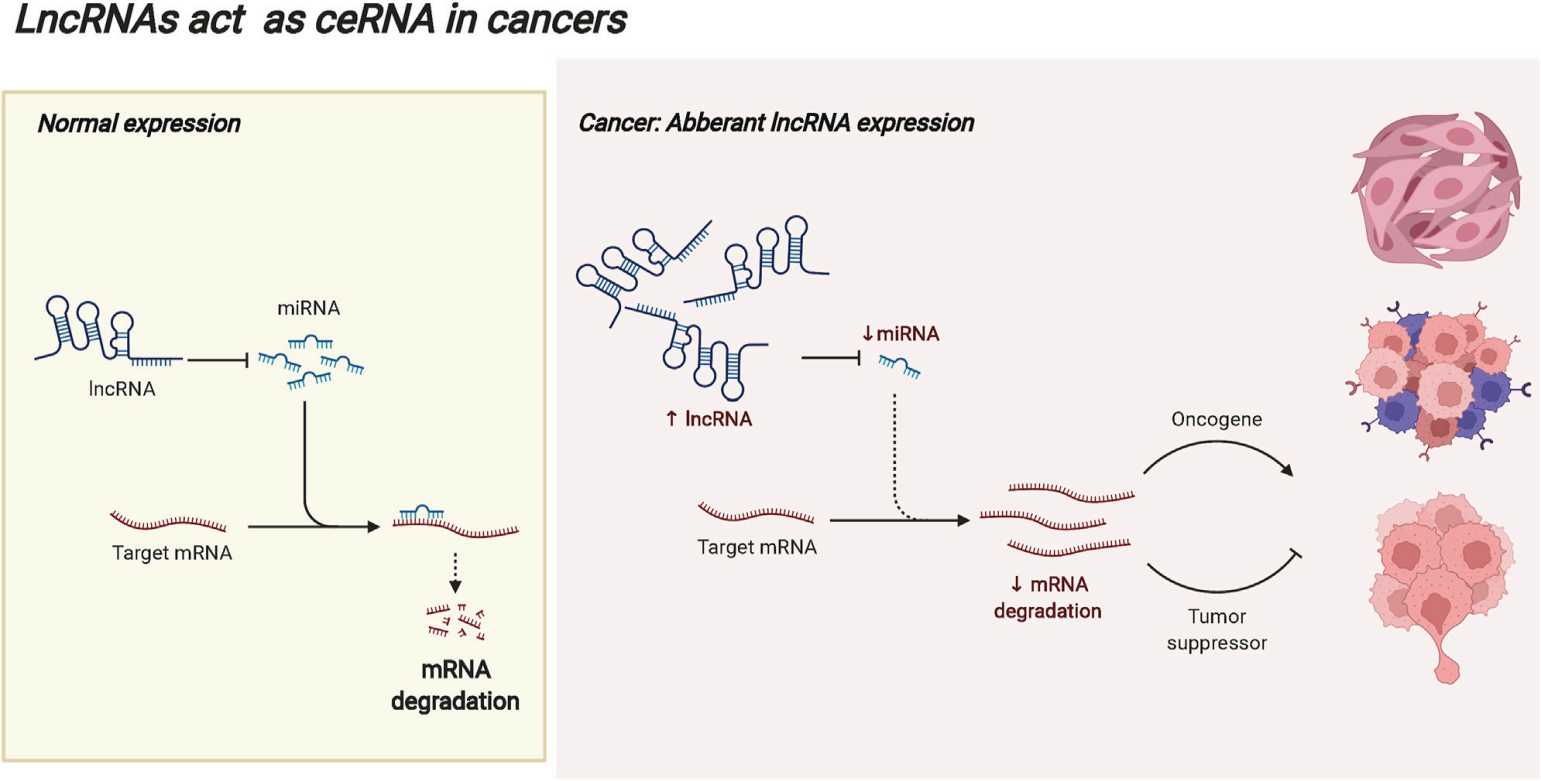
The ceRNA mechanism of lncRNAs. In the cytoplasm, lncRNAs can act as competing endogenous RNAs (ceRNAs) to compete with miRNAs for binding and contribute to the release of target mRNAs. In the occurrence and progression of tumors, abnormally expressed lncRNAs may break this balance, resulting in the abnormal expression of tumor-promoting genes or tumor suppressor genes, and promoting the malignant progression of tumors.

LncRNAs are defined as non-protein coding RNAs with transcripts longer than 200 bp (Huang et al., 2022; Yang et al., 2022), and they are considered byproducts of RNA polymerase II transcription with no biological function (Fernandes et al., 2021; Kong et al., 2021; Nojima and Proudfoot 2022). At present, there is no unified classification standard for lncRNAs (Nojima and Proudfoot 2022). According to the localization, lncRNAs are divided into cytoplasmic lncRNAs and nuclear lncRNAs, some of which are located in both the nucleus and the cytoplasm (Jianfei Tang et al., 2021). LncRNAs play different regulatory functions according to cellular localization (Cui et al., 2021). In the cytoplasm, lncRNAs act as competing endogenous RNAs (ceRNAs) to compete with miRNAs for binding and contribute to the release of target mRNAs. In tumors, abnormally expressed lncRNAs may break this balance, resulting in the abnormal expression of tumor-promoting genes or tumor-suppressor genes, and promoting the malignant progression of tumors. The ceRNA mechanism of lncRNAs is displayed in Figure 3. LncRNAs can be classified into five types according to the location in the genome relative to the protein-coding genes. The righteous lncRNAs overlap with exon regions of code-capable genes. The transcription of antisense lncRNAs begins with the reverse transcription of protein-coding genes. Bidirectional lncRNA expression start sites are very close to those of neighboring coding genes on the antisense strands. Basal lncRNAs originate from intronic regions, and intergenic lncRNAs are located in the interval between two genes on the chromosome (Cui et al., 2021; Jin et al., 2021). Based on their molecular function, lncRNAs are classified into decoy, guide, and backbone molecules. Decoy molecules are sufficient to induce transcription factors and inhibit the transcription of downstream genes. Guide molecules can bind to DNA or proteins at the same site and guide them to the site of action, enhancing the transcriptional activity of genes. The backbone molecules act as scaffolds for protein complexes, forming nucleic acid-protein complexes with target proteins and transferring the enzyme molecules involved in epigenetics to the protein complexes (Liu et al., 2022; Mercer et al., 2022). LncRNAs play critical regulatory roles in life activities and biological processes such as development, gene expression, stem cell differentiation, cell proliferation, and metastasis, indicating the close correlation to the occurrence and development of human diseases (DeSouza et al., 2021; Di Lu et al., 2021).

### 2.3 CircRNAs

**FIGURE 4:**
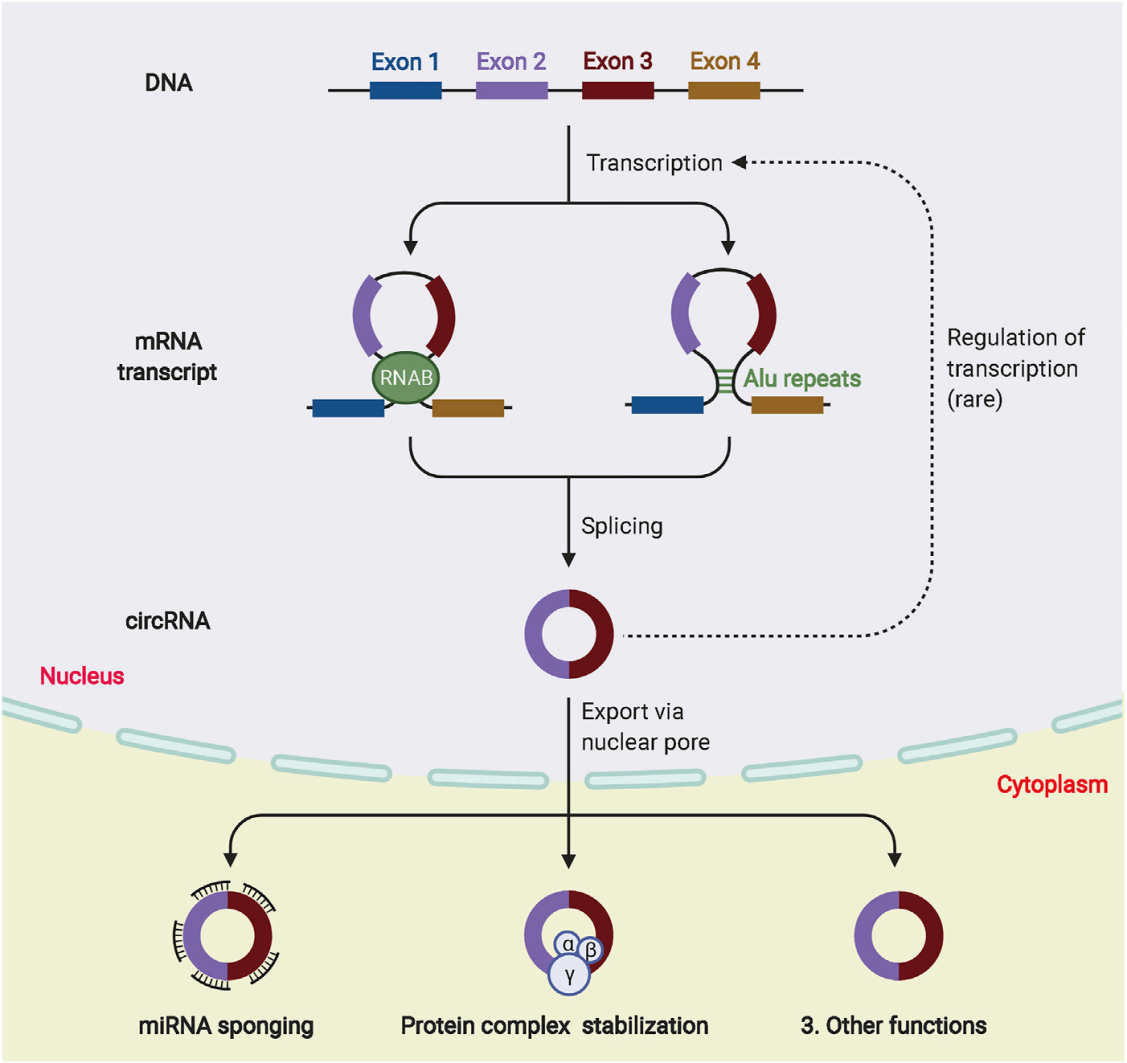
The biogenesis and biological function of circRNAs. Different circRNAs can be generated from pre-mRNA by back-splicing. According to the synthesis mechanism, circRNA can be divided into ecircRNA, ciRNA and eiciRNA. In the nucleus, circRNAs can participate in transcriptional regulation. In the cytoplasm, circRNAs are involved in many biological processes, such as miRNA sponge formation and binding to RBP.

CircRNAs are closed-loop molecules with unique structures that are widely distributed in animal and plant cells and regulate gene expression. They exist independently of proteins without being affected by exonucleases (Yue Zhang et al., 2022). Because of their low abundance and the limitation of detection technology, circRNAs were initially ignored as abnormal products of RNA splicing (Wen et al., 2022). The development of high-throughput sequencing technology has led to the identification of an increasing number of circRNAs (Kai Wang et al., 2022). Most circRNAs originate from exons in the coding regions of genes, whereas others originate from the 3′-UTR, 5′-UTR, introns, intergenic regions and antisense RNAs (Caba et al., 2021). CircRNAs are classified into four types, namely, exonic circRNAs (ecircRNAs), circular intronic RNAs (ciRNAs), exonic-intronic circRNAs (eiciRNAs), and tRNA intronic circRNAs (tricRNAs) (Wenqing Zhang et al., 2021). The most abundant and well studied circRNAs are exonucleotide circRNAs, which account for more than 80% of the total and are mainly localized in the cytoplasm. CiRNAs and eiciRNAs are abundant in the nucleus, and most circRNAs produced by different species are relatively evolutionarily conserved. The expression of the same circRNA varies greatly between diseased and non-diseased tissues and between different tissues or periods because of trans-shear (Tian Tian et al., 2021). In addition, circRNAs with covalent closed-loop structures are more stable than related linear mRNAs *in vivo* because they do not have 5′ caps or 3′ Poly A tails, which makes them highly resistant to the nucleic acid exonuclease (RNAse) (Zeng et al., 2021). Currently, there are three hypothetical models to explain the possible mechanisms of exon circRNA production, RNA binding protein (RBP)-mediated cyclization, intron pairing-driven cyclization, and lasso-driven cyclization (Kim et al., 2021; Sinha et al., 2021). The RBP connects two non-adjacent introns at both ends of the sequence, promoting the formation of a loop by bringing the two ends close together and then forming an exonic loop RNA by splicing. This model is defined as intron pairing-driven cyclization. The lasso-driven cyclization suggests that the pre-mRNA is in a half-folded state. During the transcription process, the non-adjacent exons approach each other and are driven by transacting factors to create a lasso. The intron sequence is removed by splicing in the lasso structure to form the circRNA. The biogenesis and biological function of circRNAs are shown in Figure 4 **.**

## 3 Noncoding RNAs in skin cancer

NcRNAs play an essential role in the occurrence and development of skin cancers. This review summarizes the noncoding RNA aspects related to the metastasis mechanism of skin malignancies. The information may help identify new therapeutic targets related to the mechanism of tumor metastasis and provide new opportunities to improve the survival rate and quality of life of patients with skin malignancies.

### 3.1 MiRNAs in skin cancer

Sand et al. (Sand et al., 2012) identified 16 miRNAs that are highly expressed in BCC tissues and 10 miRNAs significantly downregulated in BCC tissues compared with adjacent skin by microarray miRNA analysis. MiR-203 inhibits BCC cell proliferation, differentiation, and tumor growth by targeting c-JUN as an oncogenic factor in BCC (Sonkoly et al., 2012). MiR-451a inhibits BCC cell growth and colony formation by targeting TBX1, which may serve as an effective therapeutic target for BCC (Sun and Jiang 2018). MiR-18a expression is significantly increased in BCC tissues and cells and promotes BCC malignant progression by targeting the Akt/mTOR/Beclin 1/LC3 axis (Mi et al., 2020). MiR-17-92 cluster members and miR-143–145 cluster members are involved in BCC progression as pro-oncogenes and oncogenes, respectively (Sand et al., 2017). The miRNAs involved in BCC are listed in Table 1 **.**

**TABLE 1:** Functional characterization of miRNAs in basal cell carcinoma.

miRNAs	Expression	Function role	Related genes	References
miR-203	Down	Inhibit cell proliferation and differentiation and tumor growth	c-JUN	Sonkoly et al. (2012)
miR-451a	Down	Inhibit cell growth and colony formation	TBX1	Sun and Jiang, (2018)
miR-18a	Up	Promote tumor malignant progression	Akt/mTOR/Beclin 1/LC3	Mi et al. (2020)
miR-17-92	Up	Promote tumor malignant progression	/	Sand et al. (2017)
miR-143-145	Down	Inhibit tumor malignant progression	/	Sand et al. (2017)

Various miRNAs are upregulated in CSCC and function as oncogenes to promote the malignant behavior of CSCC (Table 2 and Table 3). MiR-365 promotes the malignant progression of CSCC by targeting nuclear factor I B (NFIB), which may serve as a potential therapeutic target (Meijuan Zhou et al., 2014). MiR-31 expression promotes the malignant progression of tumors by increasing migration, invasion, and colony formation (Shao Hua Wang et al., 2014). MiR-365b and miR-135b target leucine zipper tumor suppressor 1 in CSCC tissues to promote migration and invasiveness (Olasz et al., 2015). Increased miR-365 expression promotes the malignant progression of tumors by targeting cyclin-dependent kinase 6 (Zhou et al., 2015). MiR-346 inhibits SRC kinase signaling inhibitor 1 expression and promotes CSCC cell proliferation and migration (Chen et al., 2016). Cadherin 1 and matrix metaloproteases (MMPs) are the downstream targets of miR-199a-5p and miR-217 in CSCC tissues, and their upregulation promotes malignant progression (Wang et al., 2016). Multiple miRNAs are upregulated in CSCC and regulate various downstream signaling targets to promote tumor cell proliferation, migration, and invasion. MiR-27 regulates PTRF (Bai et al., 2017), miR-31 regulates rhobtb1 (Lin et al., 2017), miR-506 regulates laminin subunit gamma 1 (Jian Zhou et al., 2019), miR-125b regulates MMP 13 (Xu et al., 2012), miR-124/214 regulates ERK1/2 (Yamane et al., 2013), and miR-196a promotes malignant behavior in the same cellular signaling pattern (Wei Zhang et al., 2022). In addition to promoting proliferation and migration, numerous miRNAs inhibit cancer cell apoptosis to enhance the malignant behavior. MiR-365 expression is upregulated in CSCC and inhibits apoptosis by targeting BAX (Zhou et al., 2017). MiR-142-5p targets PTEN (Bai et al., 2018), miR-186 targets reticulophagy regulator 1 (Xinde Hu et al., 2019), and miR-320a regulates autophagy related 2B (Bi et al., 2021) to inhibit cancer cell apoptosis. Programmed cell death 5 is the dominant downstream target of miR-766, the upregulation of which inhibits the apoptosis of CSCC cells (Liu et al., 2020). Increased expression of miR-22 decreases CSCC chemosensitivity by targeting the Wnt/β-catenin signaling axis, thus promoting malignant progression (Yuan et al., 2021). MiR-486-3p targets flotillin 2 (FLOT2) to promote CSCC cell proliferation, migration, and tumorigenicity (Li et al., 2022). Angiogenesis is a common antineoplastic target for drugs and ncRNAs. MiR-361-5p inhibits SCC cell proliferation and angiogenesis by targeting VEGFA (Kanitz et al., 2012).

**TABLE 2:** Functional characterization of upregulated miRNAs in cutaneous squamous cell carcinoma.

miRNAs	Expression	Function role	Related genes	References
miR-365	Up	Promote tumor malignant progression	NFIB/CDK6/BAX	(Meijuan Zhou et al., 2014; Zhou et al., 2015)
miR-31	Up	Promote cell migration, invasion, and colony formation	RhoBTB1	(Aoxue Wang et al., 2014; Lin et al., 2017)
miR-135b	Up	Increase the motility and aggressiveness of cancer cells	LZTS1	Olasz et al. (2015)
miR-346	Up	Promote cell proliferation and migration	SRCIN1	Chen et al. (2016)
miR-199a-5p	Up	Promote tumor malignant progression	CDH1/MMPs	Wang et al. (2016)
miR-217	Up	Promote cell growth, cell cycle and invasion	PTRF	Bai et al. (2017)
miR-142-5p	Up	Promote cell growth and inhibit cell apoptosis	PTEN	Bai et al. (2018)
miR-506	Up	Promote cell viability, migration, and invasion	LAMC1	Wu Zhou et al. (2019)
miR-186	Up	Promote cell growth and inhibit cell apoptosis	RETREG1	Yan Hu et al. (2019)
miR-766	Up	Promote cell proliferation, migration, invasion and inhibit cell apoptosis	PDCD5/MMPs	Liu et al. (2020)
miR-320a	Up	Promote cell growth and inhibit cell apoptosis	ATG2B	Bi et al. (2021)
miR-22	Up	Promote chemosensitivity	Wnt/β-catenin	Yuan et al. (2021)
miR-486-3p	Up	Promote cell proliferation, migration and tumorigenicity	FLOT2	Li et al. (2022)
miR-196a	Up	Promote cell proliferation, migration, invasion	—	Wei Zhang et al. (2022)

**TABLE 3:** Functional characterization of downregulated miRNAs in cutaneous squamous cell carcinoma.

miRNAs	Expression	Function role	Related genes	References
miR-125b	Down	Inhibit proliferation, colony formation, migration and invasion	MMP13	Xu et al. (2012)
miR-124/-214	Down	Inhibit cell proliferation	ERK1/2	Yamane et al. (2013)
miR-361-5p	Down	Inhibit cell invasion and angiogenesis	VEGFA	Kanitz et al. (2012)
miR-20a	Down	Suppress cell proliferation and metastasis	LIMK1	Jianda Zhou et al. (2014)
miR-199a	Down	Inhibit tumor growth and metastasis	CD44	Shao Hua Wang et al. (2014)
miR-199a-5p	Down	Inhibit cell invasion	BCAM, FZD6 and DDR1	Kim et al. (2015)
miR-148a	Down	Inhibit cell proliferation and metastasis	MAP3K4 and MAP3K9	Bai et al. (2018)
miR-204	Down	Inhibit tumor malignant progression	PTPN11	Toll et al. (2016)
miR-497	Down	Inhibit tumor malignant progression	SERPINE-1	Mizrahi et al. (2018)
miR-497	Down	Inhibit cell proliferation	FAM114A2	Wei et al. (2018)
miR-3619-5p	Down	Inhibit cell proliferation and cisplatin resistance	KPNA4	Zhang et al. (2019)
miR-30a-5p	Down	Inhibit tumor malignant progression	FOXG1	Zhang et al. (2019)
miR-199a-5p	Down	Inhibit tumor malignant progression	Sirt1/CD44ICD	Lu et al. (2020)
miR-27a	Down	Inhibit tumor malignant progression	EGFR	Yinghui Wang et al. (2019)
miR-125b	Down	Inhibit cell proliferation and promote cell apoptosis	STAT3	Ke Tian et al. (2020)
miR-216b	Down	Inhibit tumor malignant progression	TPX2	Feng et al. (2020)
miR-10a	Down	Inhibit tumor malignant progression	SDC-1	Xiong et al. (2020)
miR-214	Down	Inhibit tumor malignant progression	VEGFA and Bcl-2	Ma et al. (2020)
miR-451a	Down	Inhibit tumor malignant progression	PDPK1	Fu et al. (2021)
miR-130a	Down	Inhibit tumor malignant progression	ACVR1	Lohcharoenkal et al. (2021)
miR-573	Down	Inhibit cell proliferation, migration and invasion	PICSAR	Kai Wang et al. (2022)
miR-30c	Down	Inhibit cell proliferation and cisplatin resistance	SIRT1	Liu et al. (2022)

Decreased expression of various miRNAs results in CSCC suppression. MiR-20a is downregulated in CSCC tissues and inhibits cell proliferation and metastasis by targeting LIM domain kinase 1 (Jianda Zhou et al., 2014). Decreased expression of miR-199a affects the interaction between CD44 and Ezrin and inhibits CSCC proliferation and metastasis (Kanitz et al., 2012). MiR-199a-5p targets BCAM, FZD6, and DDR1 (Kim et al., 2015), and miR-148a targets MAP3K4 and MAP3K9 to inhibit the proliferation, invasiveness, and metastasis of CSCC cells (Luo et al., 2015). MiR-204 is downregulated in CSCC tissues and regulates protein tyrosine phosphatase non-receptor type 11 (PTPN11), modulating the signal transducer and activator of transcription 3 (STAT3) and MAPK signaling pathways to inhibit the malignant progression of CSCC (Toll et al., 2016). MiR-497 expression is decreased in CSCC tissues and inhibits the malignant progression of CSCC by targeting PTPN11. Decreased expression of miR-497 inhibits the malignant progression of CSCC by targeting PTPN11 and SERPINE-1 in the AKT/mTOR and EMT processes (Mizrahi et al., 2018) and the FAM114A2 signaling pathway (Wei et al., 2018). MiR-3619-5p is downregulated in CSCC and modulates karyopherin subunit alpha four to inhibit proliferation and cisplatin resistance (Zhang et al., 2019). MiR-30a-5p (Shao et al., 2019) and miR-199a-5p are downregulated in CSCC tissues and modulate forkhead box G1 (FOXG1) to inhibit CSCC cell migration, invasion, and colony formation. MiR-199a-5p also inhibits the migration and tumorigenicity of CSCC by targeting Sirt1/CD44ICD (Lu et al., 2020). MiR-27a regulates EGFR (Yinghui Wang et al., 2019), miR-125b regulates STAT3 (Ke Tian et al., 2020), miR-216b regulates TPX2 (Feng et al., 2020), and miR-10a regulates SDC-1 (Xiong et al., 2020) to inhibit CSCC cell proliferation, migration, and invasion. MiR-214 is downregulated in CSCC tissues and inhibits migration and invasion by regulating VEGFA, Bcl-2, and the Wnt/β-catenin pathway (Feng et al., 2020). Downregulation of miR-451a and miR-130a inhibits CSCC cell proliferation, migration, invasion, and EMT by regulating the PI3K/AKT signaling pathway through 3-phosphoinositide dependent protein kinase 1 (Fu et al., 2021). Decreased miR-130a expression inhibits CSCC proliferation, cell motility, and invasion by regulating the ACVR1 and BMP/SMAD pathways (Lohcharoenkal et al., 2021). MiR-573 and miR-30c are downregulated in CSCC tissues and inhibit proliferation, migration, and invasion by modulating PICSAR (Lipeng Wang et al., 2022) and SIRT1 (Liu et al., 2022).

In summary, miRNAs play an essential role in the development and progression of CMM, promoting or inhibiting malignant progression by regulating cell proliferation, invasion, metastasis, drug resistance, the immune microenvironment, cell cycle progression, and apoptosis. Detailed information is provided in Table 4, Table 5, Table 6.

**TABLE 4:** Functional characterization of miRNAs in cell proliferation, cell cycle and apoptosis of melanoma.

Cellular process	miRNAs	Related genes	References
Cell proliferation	miR-377	E2F3 and MAP3K7	Zehavi et al. (2015)
	miR-664	PLP2	Ding et al. (2015)
	miR-21	PTEN	Saldanha et al. (2016)
	miR-330-5p	TYR and PDIA3	Su et al. (2016)
	miR-4262	KLF6	Zhang et al. (2016)
	miR-135b	LATS2	Xinde Hu et al. (2019)
	miR-181	STAT3-AKT	He et al. (2020a)
	miR-140-3p	ABHD2	He et al. (2020b)
	miR-34a	ZEB1	Xu et al. (2021)
Cell cycle	miR-193b	CCND1	Chen et al. (2010)
	miR-186	—	Su et al. (2018)
	miR-21-5p	CDKN2C	Yang et al. (2020)
Cell apoptosis	miR-125b	—	(Glud et al., 2011; Nyholm et al., 2014)
	miR-92a-3p	MYCBP2	Venza et al. (2016)
	miR-365	BCL2	Zhu et al. (2018)
	miR-128	CCL18	Song et al. (2018)

**TABLE 5:** Functional characterization of miRNAs in cell migration and metastasis of melanoma.

Cellular process	miRNAs	Related genes	References
Migration	miR-532-5p	RUNX3	Kitago et al. (2009)
	MiR-125b	/	Glud et al. (2010)
	miR-200 and miR-203	E-cadherin	van Kempen et al. (2012)
	miR-21	TIMP3	Martin del Campo et al. (2015)
	miR-21	PDCD4	Jiao et al. (2015)
Metastasis	miR-23a	ATG12	Guo et al. (2017)
	miR-367	PTEN	Long et al. (2018)
	miR-509-3p	GPC6	Li et al. (2019)
	miR-152-5p	TXNIP	Li et al. (2020)

**TABLE 6:** Functional characterization of miRNAs in drug resistance and the tumor microenvironment of melanoma.

Cellular process	miRNAs	Related genes	References
Drug resistance	miR-514a	NF1	Stark et al. (2015)
	miR-579-3p	Verofini	Wang et al. (2018)
	miR-26a	HMGB1- dabrafenib	Yan Yu et al. (2019)
	miR-205	INPPL1	Sánchez-Sendra et al. (2020)
	miR-92a-3p	Dasatinib	Yuxiong Jiang et al. (2021)
Tumor microenvironment	miR-125b-5p	LIPA	Gerloff et al. (2020)

### 3.2 LncRNAs in skin cancer

Sand et al. (2016a) identified 1851 upregulated lncRNAs and 2165 downregulated lncRNAs in microarrays of BCC compared with non-lesioned skin. Further exploration demonstrated the biological roles and molecular mechanisms of these differentially expressed lncRNAs. Lnc-PICSAR is upregulated in DDP-resistant CSCC cells and regulates the miR-485-5p/REV3-like DNA directed polymerase zeta catalytic subunit (REV3L) signaling axis (Wang et al., 2020). LINC00963 is increased in CSCC tissues and promotes malignant progression by upregulating SOX4 expression through the uptake of miR-1193 (Jingwen Wang et al., 2019). LINC00641 is downregulated in CSCC cell lines and inhibits CSCC growth and metastasis by downregulating miR-424 expression (Quan Liu et al., 2021). LINC00162 is specifically expressed in CSCC but not in keratin-forming cells in normal skin. Overexpression of LINC00162 promotes CSCC tumorigenesis *in vitro* and *in vivo* (Piipponen et al., 2016). LINC00319 is increased in CSCC and inhibits apoptosis, promoting cell proliferation, cell cycle progression, cell migration, and invasion. Mechanistic studies indicate that LINC00319 may exert an oncogenic function in CSCC by binding to miR-1207-5p and promoting the expression of cyclin-dependent kinase 3 (Li et al., 2018). LINC00520 regulates EGFR expression and inactivates the PI3K/Akt pathway, thereby inhibiting the development of CSCC (Mei and Zhong, 2019). USF1 activates LINC01048 to promote CSCC proliferation and inhibit apoptosis by interacting with TATA-box binding protein associated factor 15 (TAF15) to upregulate YAP1 (Chen et al., 2019). ALA-PDT promotes TINCR expression through the ERK1/2-SP3 pathway to induce apoptosis and autophagy, leading to the malignant progression of CSCC (Xiaobo Zhou et al., 2019). HOX transcript antisense RNA (HOTAIR) is elevated in CSCC cells and promotes migration, proliferation, and EMT, possibly by binding to miR-326 and promoting PRA1 domain family member 2 (PRAF2) expression (Guo Jun Yu et al., 2019). HOTAIR promotes CSCC stemness and progression by upregulating Sp1 and modulating miR-199a (Chen et al., 2022). LINC00346 is highly expressed in CSCC tissues to promote CSCC malignant progression by activating STAT3 and MMP expression (Piipponen et al., 2020). Small Cajal body-specific RNA 2 is upregulated in CSCC tissues and promotes proliferation and invasion by suppressing miR-342-3p expression (Zhang et al., 2020a). PICSAR functions as an oncogene by regulating the miR-125b/YAP1 signaling axis, and EZR-AS1 regulates the PI3K/AKT signaling pathway to promote CSCC cell proliferation and invasion and inhibit apoptosis (Qirong Lu et al., 2021). Overexpression of H19 promotes malignant behavior and induces apoptosis. H19 promotes the expression of the EMT-related marker miR-675 and inhibits p53 expression (Shunhao Zhang et al., 2021). Hcp5 can upregulate EZH2 expression by competitively binding to miR-138-5p, promoting autophagy and suppressing apoptosis by regulating the STAT3/VEGFR2 pathway, leading to malignant progression of CSCC (Zou et al., 2021). Neat1 promotes autophagy and decreases apoptosis by binding to miR-361-5p, promoting CSCC proliferation and invasion by activating the Wnt pathway (Shiqiu Jiang et al., 2021). NEAT1 promotes CSCC proliferation, invasion, and metastasis by regulating the expression of metalloplasmic proteins (Gong et al., 2022).

FOXD2-AS1 expression is increased in cutaneous melanoma tissue specimens and cell lines (Ren et al., 2019), inhibiting proliferation, migration, and invasion of cutaneous melanoma cells by regulating phospho-Akt expression (Wu Zhou et al., 2019). CPS1-IT1 inhibits melanoma metastasis by competitively binding to BRG1 and impairing CYR61 expression. Linc00961 is decreased in cutaneous melanoma tissues and functions as a ceRNA to regulate the miR-367/PTEN axis and inhibit cell proliferation and invasion (Mu et al., 2019). LncRNA FOXD3-AS1 promotes the proliferation, invasion, and migration of CMM by binding to miR-325 and promoting MAP3K2 expression as a potential cause of cutaneous melanoma (Wei et al., 2019). FENDRR suppresses MMP2 and MMP9 and antagonizes the JNK/c-Jun pathway to promote proliferation, migration, and invasion of melanoma (Shuang Chen et al., 2020). LncRNA-TTN-AS1 promotes TTN transcription and increases the stability of TTN mRNA thus inducing tumor progression. LINC00518 is upregulated in CMM and induces radioresistance of CMM by regulating glycolysis through a miR-33a-3p/HIF-1α negative feedback loop (Yan Liu et al., 2021). LINC01116 promotes cell proliferation, migration, invasion and EMT to sponge miR-3612 by regulating GDF11 and SDC3, thereby promoting melanoma progression (Yanhua Wang et al., 2022). LncRNA Tincr is downregulated in CMM, which promotes CTGF, CCN1, and AXL expression, leading to cell proliferation, invasion, and apoptosis inhibition in CMM cell lines. p53 upregulates PURPL in melanoma to promote cell proliferation, colony formation, migration, and invasion, and inhibiting apoptosis (Shuo Han et al., 2021). Mechanistic studies show that PURPL promotes mTOR-mediated phosphorylation of ULK1 at Ser757 by physically interacting with mTOR and ULK1, limiting the autophagic response to suppress apoptosis. TEX41 is activated by IRF4 and binds to miR-103a-3p to upregulate C1QB, thereby promoting cell proliferation, migration, and invasion while inhibiting apoptosis, suggesting that TEX41 is a potential therapeutic target for melanoma (Zheng et al., 2021). Linc00518 is upregulated in CMM tissues and promotes malignant progression via the miR-526b-3p/EIF5A2 axis (Xu et al., 2022).

Taken together, these data indicate that lncRNAs function as tumor-promoters or tumor-suppressors in CMM by interacting with miRNAs and regulating protein expression.

### 3.3 CircRNAs in skin cancer

The detailed information of circRNAs in skin cancers is shown in Table 7.

**TABLE 7:** Functional characterization of circRNAs in skin cancers.

Cancer	circRNAs	Expression	Function role	Related genes	References
Basal cell carcinoma	Circ_0005795	Up	Promote cell viability, colony formation, and suppress cell apoptosis	miR-1231 and caspase-3	Yating Li et al. (2021)
	Circ_NCKAP1	Up	Promote cell proliferation, inhibit cell apoptosis	miR-148b-5p/HSP90 axis	Fan et al. (2021)
Malignant melanoma	circRNA_0084043	Up	promote melanoma cell proliferation, invasion and migration	miR-153-3p/Snail axis	Luan et al. (2018)
	hsa_circ_0025039	Up	promote cell proliferation, colony formation ability, invasion and glucose metabolism in melanoma cells	miR-198/CDK4	Bian et al. (2018)
	circMTUS1	Up	promote cell proliferation	hsa-miR-622/hsa-miR-1208	Shang et al. (2019)
	Circular RNA ITCH	Up	downregulate GLUT1 and suppresses glucose uptake in melanoma to inhibit cancer cell proliferation	GLUT1	Lin et al. (2021)
	circMYC	Up	promote the proliferation of human melanoma cells and Mel-CV cells. repress Mel-CV cell glycolysis and LDHA activities	Mel-CV cells/miR-1236/c-MYC-SRSF1 axis	Jin et al. (2020)
	CDR1as	Down	inhibit malignant progression	miR-7/LINC00632/IGF2BP3	Hanniford et al. (2020)
	circ_0084043	Up	promote cell proliferation, migration and invasion, facilitate apoptosis in A375 and SK-MEL-28 cells	miRNA (miR)-429/TRIB2	Zhibing Chen et al. (2020)
	circ-FOXM1	Up	promote cell proliferation, invasion, and glycolysis and facilitated cell apoptosis	miR-143-3p/FLOT2/MTT assay	Ke Tian et al. (2020)
	circ_0002770	Up	promote cell invasion, migration, and proliferation	miR-331-3p/DUSP5 and TGFBR1	Qian et al. (2020)
	circ_0020710	Up	promoted melanoma cell proliferation, migration and invasion	miR-370-3p/CXCL12	Wei et al. (2020)
	circ 0001591	Up	promoted cell growth and cell invasion and reduced apoptotic rate of melanoma	ROCK1/PI3K/AKT/ROCK1/miR-431-5p	Yin et al. (2021)
	circ_0079593	Up	Promotes cell proliferation, cell cycle progression, migration, invasion, inhibits cell apoptosis, and promotes tumor growth	miR-573/ABHD2	Zhao et al. (2021)
	CircRNA_0082835	Up	Promotes proliferation, invasion and migration of melanoma cells and regulates cell cycle levels	EZH2/miR-429	Sun et al. (2021)
	circZNF609	Up	Promotes the invasion, migration and proliferation of melanoma cells and inhibits apoptosis	miR-138-5p/SIRT7 axis	Yan Liu et al. (2021)
	circVANGL1	Up	Promotes proliferation, migration and invasion of melanoma cells	miR-150-5p/TGF-β	Zhou et al. (2021)
Cutaneous squamous cell carcinoma	circ_0070934	Up	associated with tumor aggressiveness	miR-1238/miR-1247–5p	An et al. (2019)
	hsa_circ_0070934	Up	Promotes the invasion and proliferation potential of CSCC cells and inhibits apoptosis	miR-1236-3p/HOXB7	Zhang et al. (2020a)
	circPVT1	Up	Promote CSCC cell proliferation and migration	/	Shuang Chen et al. (2020)
	circRNA_001937	Up	Promote CSCC progression and inhibit apoptosis	miRNA-597-3p/FOSL2	Gao et al. (2020)
	circSEC24A	Up	Promotes cell proliferation, migration, invasion and glycolysis, inhibits apoptosis	miR-1193/MAP3K9 axis	Xiaoyan Lu et al. (2021a)
	hsa_circ_0001360	Down	Inhibits the proliferation, migration and invasion of SCL-1 cells and promotes apoptosis	/	Chen et al. (2021)
	Circ_0067772	Up	Promotes proliferation, migration and invasion of CSCC cells	miR-1238-3p/FOXG1 axis	Ziwei Li et al. (2021)
	hsa_circ_0070934	Up	Promote CSCC cell proliferation, cell cycle process, migration, invasion, and inhibit apoptosis	miR-136-5p/PRAF2 axis	Xiong et al. (2021)
	circ-CYP24A1	Up	Promote cell proliferation, migration and invasion, inhibit apoptosis	CDS2、MAVS 和 SOGA	Zhang et al. (2021a)
	hsa_circ_0008234	Up	Increased the cell viability and colony formation of cSCC cells	miR-127-5p/ADCY7	Cai et al. (2021)
	circFADS2	Down	Inhibits CSCC cell proliferation, metastasis and glycolysis	miR-766-3p/HOXA9	Zhang et al. (2021b)
	circ-LARP1B	Up	Promotes cell viability, colony-forming ability, migration, invasion, cell cycle progression and glycolysis of CSCC cells, and inhibits apoptosis	miR-515-5p/TPX2 axis	Yan Wang et al. (2022)
	circ_0001821	Up	Promotes cell viability, colony formation, cell cycle progression and metastasis, and inhibits apoptosis *in vitro* and promotes tumor growth *in vivo*	miR-148a-3p/EGFR axis, PI3K/Akt pathway	Yue Zhang et al. (2022)

A circRNA microarray identified 23 upregulated and 48 downregulated circRNAs in BCC with a potential function in regulating the development of BCC. However, the underlying mechanism needs to be examined (Sand et al., 2016b). Circ_0005795 expression is increased in BCC tissues, and knockdown of circ_0005795 inhibits cell viability, colony formation, and anti-apoptotic protein levels while increasing caspase-3 activity (Yating Li et al., 2021). Circ_0005795 exerts its pro-tumor effects by binding to miR-1231. Circ_NCKAP1 promotes the malignant progression of cutaneous BCC by regulating the miR-148b-5p/HSP90 signaling axis (Fan et al., 2021).

Circ_0070934 is expressed aberrantly in CSCC. Overexpression of circ_0070934 promotes cell proliferation, invasion, and migration, and inhibits apoptosis (An et al., 2019). The hsa_circ_0070934 binds to miR-1236-3p and regulates homeobox B7 expression to promote the malignant progression of CSCC (Zhang et al., 2020b). GSPS downregulates the expression of hsa_circ_0070934 and inhibits CSCC cell proliferation, cell cycle progression, migration, and invasion, and promotes apoptosis. However, GSPS also plays an oncogene role in CSCC by regulating the hsa_circ_0070934/miR-136-5p/PRA1 domain family member 2 (PRAF2) axis (Xiong et al., 2021). CircRNA_001937 promotes the malignant progression of CSCC by regulating the miR-597-3p/FOSL2 pathway as determined through *ex vivo* assays (Gao et al., 2020). CircSEC24A and MAP3K9 are upregulated in CSCC tissues, whereas the expression of miR-1193 is decreased (Ziwei Lu et al., 2021). Inhibition of circSEC24A inhibits malignant behavior and glycolysis to induced apoptosis, whereas overexpression of circSEC24A promotes tumor growth. CircSEC24A may act as a molecular sponge for miR-1193 and thus regulate the expression of MAP3K9 (Chen et al., 2021). The hsa_circ_0001360 inhibits cell proliferation, migration, and invasion and promotes apoptosis, acting as an antioncogene in CSCC. Circ_0067772 is upregulated in CSCC tissues and cells, and overexpression of circ_0067772 regulates FOXG1 by binding to miR-1238-3p, thereby promoting cell proliferation, migration, and invasion. Inhibition of circ_0067772 suppresses tumor growth (Xiaoqing Li et al., 2021).

Zhang et al. (2021c) also screened differentially expressed circRNAs in CSCC exosomes by RNA-seq analysis and identified 25 upregulated and 76 downregulated exosomal circRNAs. Circ-CYP24A1 promotes CSCC malignant progression by inducing the expression of CDS2, MAVS, and SOGA. Hsa_circ_0008234 promotes CSCC malignant progression by targeting miR-127-5p to regulate adenylate cyclase 7 expression (Cai et al., 2021). Circfads2 is downregulated in CSCC tissues and inhibits CSCC cell proliferation, metastasis, and glycolysis (Wenqing Zhang et al., 2021). Mechanistic studies suggest that circfads2 regulates the miR-766-3p/HOXA9 axis to inhibit CSCC progression, and is thus a potential therapeutic target for CSCC. Circ-LARP1B/miR-515-5p/TPX2 microtubule nucleation factor regulatory axis is involved in the malignant progression of CSCC by promoting cell viability, colony formation, migration, invasion, cell cycle progression, and glycolysis and inhibiting apoptosis (Yan Wang et al., 2022). Circ_0001821 plays a pro-oncogenic role in CSCC development by regulating the miR-148a-3p/EGFR signaling axis and PI3K/Akt pathway (Yue Zhang et al., 2022).

Luan et al. (Luan et al., 2018) found that Circ_0084043 is upregulated in melanoma tissues and functions as a sponge for miR-153-3p to upregulate Snail expression and thus promote melanoma cell proliferation, invasion, and migration. The hsa_circ_0025039 promotes CDK4 expression by binding to miR-198, promoting cell proliferation, colony-forming ability, invasion, and glucose metabolism (Bian et al., 2018). Circ_0084043 expression is increased in melanoma tissues and promotes the malignant development of melanoma by regulating the miR-429/TRIB2 signaling axis and Wnt/β-catenin signaling pathway (Xu E Chen et al., 2020). Circ-FOXM1 upregulates FLOT2 by binding to miR-143-3p, promoting cell proliferation, invasion, and glycolysis, playing a tumorigenic role *in vivo* (Shan Tian et al., 2020). The circRNA circ_0002770 promotes dual specificity phosphatase 5 and transforming growth factor beta receptor 1 expression by binding to miR-331-3p, thereby promoting the proliferation, invasion, and migration of melanoma cells, leading to malignant tumor progression (Qian et al., 2020). Wei et al. (Wei et al., 2020) showed that circ_0020710 expression is increased in melanoma tissues and promotes proliferation, migration, and invasion. increased circ_0020710 upregulates CXCL12 expression by sponging miR-370-3p, which is associated with cytotoxic lymphocyte depletion. Circ 0001591 expression is increased in melanoma patient sera, which promotes cell growth and invasion and inhibits apoptosis (Yin et al., 2021). Circ 0001591 upregulates ROCK1 expression by binding to miR-431-5p and promotes malignant progression of melanoma by regulating the ROCK1/PI3K/AKT signaling pathway. Circ_0079593 regulates ABHD2 expression through miR-573 and promotes cell proliferation, migration, and invasion and inhibits apoptosis (Zhao et al., 2021). Circ_0082835 is upregulated in melanoma tissues and binds to miR-429 to promote the proliferation, invasion, and migration of melanoma cells, as well as regulating cell cycle progression (Sun et al., 2021). CircZNF609 inhibits DNA damage and promotes the malignant progression of melanoma by regulating the miR-138-5p/SIRT7 signaling axis (Quan Liu et al., 2021). The expression of circVANGL1 is increased in melanoma tissues and cell lines, and overexpression of circVANGL1 promotes the proliferation, migration, and invasion of melanoma cells (Zhou et al., 2021). Taken together, these findings indicate that lncRNAs function as tumor-promoters or tumor-suppressors involved in the malignancy of cutaneous melanoma through their ceRNA activity for miRNAs.

## 4 Future prospects and conclusion

Various miRNAs are involved in the regulation of skin cancer development and metastasis. The differential expression of miRNAs at different stages of skin cancer provides a new basis and novel direction for developing biomarkers and therapeutic targets. Further studies on the mechanism underlying the function of miRNAs will facilitate the development of new therapeutic strategies for the treatment of skin cancers, which will improve the outcomes of patients with advanced skin cancer. LncRNAs are critical epigenetic factors that regulate the development, progression, metastasis, and resistance to therapeutic agents in skin cancers. Although the majority of lncRNAs are expressed at low levels, the value of lncRNAs as early diagnostic, predictive, and therapeutic targets for skin cancer is becoming increasingly evident. The complexity of lncRNAs increases the difficulty of their biological investigation because the same lncRNAs function differently in distinct tumor types. Exploring the functions of lncRNAs and elucidating the molecular regulatory mechanisms involved in the development and progression of skin cancer is a new research direction.

CircRNAs have drawn increased attention in life science and medical research. CircRNAs competitively bind to miRNAs and regulate multiple tumor signaling pathways, suggesting the potential of circRNAs as biomarkers for detecting related diseases. Research on circRNAs in tumors is still in its infancy, with only a fraction of functional circRNAs available. The current circRNA database is incomplete, and there are no databases of circRNAs related to tumor prognosis for functional prediction after RNA sequencing. In addition, the mechanism of circRNAs in tumorigenesis remains to be fully elucidated. Their interaction with mRNA and its diagnostic applications show potential for clinical practice. CircRNAs show great potential in tumor diagnosis, prognosis, and treatment with high clinical value. We believe that continuing efforts and practical investigation will clarify the value of circRNAs for precision medicine in clinical diagnosis and treatment. The cross-talk of ncRNAs is complex and highly tissue specific. Therefore, exploring their functions in many cell types or tissues is essential to determine specificity and therapeutic effects. Despite the challenges associated with the application of ncRNAs, extensive research efforts have been devoted to extend investigation to ncRNA clinical trials.

This review summarized the mechanisms underlying the role of ncRNAs in the occurrence, metastasis, and drug resistance of skin cancers. Their potential application for the early diagnosis, prognosis assessment, and targeted therapy has been suggested. Several ncRNAs have undergone different phases of clinical trials. For example, a miR-122-related drug (miravirsen) and ncRNA-associated nanoparticles have been the subject of clinical trials (Gebert et al., 2014). The miRNA mimic MIRX34 was used in a phase I trial in patients with primary liver cancer (Bouchie 2013). Therefore, further exploration of clinical biosignatures and functional cell assays is needed. In addition, non-invasive methods should be considered for ncRNA harvesting.

## Glossary

BCC: basal cell carcinoma
CSCC: cutaneous squamous cell carcinoma
CM: cutaneous melanoma
miRNAs: microRNAs
lncRNAs: long non-coding RNAs
circRNAs: circular RNAs
BRAF: B-Raf proto-oncogeneserine/threonine kinase
ncRNA: non-coding RNA
mRNA: messenger ribonucleic acid
ciRNA: circular intronic RNA
eiciRNA: exon-intron circular RNA
RBP: RNA binding protein
TBX1: T-box transcription factor 1
NFIB: nuclear factor I B
LZTS1: leucine zipper tumor suppressor 1
CDK6: cyclin dependent kinase 6
SRCIN1: SRC kinase signaling inhibitor 1
CDH1: cadherin 1
LAMC1: laminin subunit gamma 1
RETREG1: reticulophagy regulator 1
PDCD5: programmed cell death 5
ATG2B: autophagy related 2B
FLOT2: flotillin 2
LIMK1: LIM domain kinase 1
STAT3: signal transducer and activator of transcription 3
PTPN11: protein tyrosine phosphatase non-receptor type 11
FAM114A2: family with sequence similarity 114 member A2
KPNA4: karyopherin subunit alpha 4
FOXG1: forkhead box G1
TPX2TPX2: TPX2 microtubule nucleation factor
PDPK1: 3-phosphoinositide dependent protein kinase 1
REV3L: REV3 like, DNA directed polymerase zeta catalytic subunit
TAF15: TATA-box binding protein associated factor 15
HOTAIR: HOX transcript antisense RNA
PRAF2PRAF2: PRA1 domain family member 2
SCARNA2: small Cajal body-specific RNA 2
MAP3K2: mitogen-activated protein kinase kinase kinase 2
PURPL: p53 level regulator
HOXB7: homeobox B7
PRAF2PRAF2: PRA1 domain family member 2
TPX2TPX2: TPX2 microtubule nucleation factor
DUSP5: dual specificity phosphatase 5
TGFBR1: transforming growth factor beta receptor 1
